# A Genome-Wide RNAi Screen for Enhancers of a Germline Tumor Phenotype Caused by Elevated GLP-1/Notch Signaling in *Caenorhabditis elegans*

**DOI:** 10.1534/g3.120.401632

**Published:** 2020-10-19

**Authors:** Diana Dalfó, Yanhui Ding, Qifei Liang, Alex Fong, Patricia Giselle Cipriani, Fabio Piano, Jialin C. Zheng, Zhao Qin, E. Jane Albert Hubbard

**Affiliations:** * Skirball Institute of Biomolecular Medicine, Departments of Cell Biology and Pathology, NYU Grossman School of Medicine; † School of Medicine, Tongji University; ‡ Center for Genomics and Systems Biology, Department of Biology, New York University and Center for Genomics and Systems Biology, New York University Abu Dhabi; § Key Laboratory of Spine and Spinal Cord Injury Repair and Regeneration of Ministry of Education, Orthopedic Department of Tongji Hospital, School of Medicine, Tongji University

**Keywords:** Pro phenotype, latent niche, synthetic tumor formation, Notch

## Abstract

Stem cells are tightly controlled *in vivo*. Both the balance between self-renewal and differentiation and the rate of proliferation are often regulated by multiple factors. The *Caenorhabditis elegans* hermaphrodite germ line provides a simple and accessible system for studying stem cells *in vivo*. In this system, GLP-1/Notch activity prevents the differentiation of distal germ cells in response to ligand production from the nearby distal tip cell, thereby supporting a stem cell pool. However, a delay in germline development relative to somatic gonad development can cause a pool of undifferentiated germ cells to persist in response to alternate Notch ligands expressed in the proximal somatic gonad. This pool of undifferentiated germ cells forms a proximal tumor that, in adulthood, blocks the oviduct. This type of “latent niche”-driven proximal tumor is highly penetrant in worms bearing the temperature-sensitive weak gain-of-function mutation *glp-1**(**ar202**)* at the restrictive temperature. At the permissive temperature, few worms develop tumors. Nevertheless, several interventions elevate the penetrance of proximal tumor formation at the permissive temperature, including reduced insulin signaling or the ablation of distal-most sheath cells. To systematically identify genetic perturbations that enhance proximal tumor formation, we sought genes that, upon RNAi depletion, elevate the percentage of worms bearing proximal germline tumors in *glp-1**(**ar202**)* at the permissive temperature. We identified 43 genes representing a variety of functional classes, the most enriched of which is “translation”. Some of these genes also influence the distal germ line, and some are conserved genes for which genetic interactions with Notch were not previously known in this system.

Stem cells are capable of self-renewal and of producing cells that differentiate. They play a vital role in the growth, homeostasis, and repair of tissues in multicellular organisms (Weissman 2000). To meet these needs, stem cells are highly adaptive to physiological and environmental changes and their behavior is tightly controlled *in vivo*. Imbalances between self-renewal and differentiation can cause deleterious conditions such as tissue degeneration and cancer (He *et al.* 2009). In many stem cell systems, the proliferation *vs.* differentiation cell fate decision of stem cells is controlled via interaction with a local microenvironment, the stem cell niche. Several signaling pathways have been implicated in various stem cell systems, including the highly conserved Notch signaling pathway (Koch *et al.* 2013).

The *C. elegans* hermaphrodite germ line provides a simple and accessible system for studying stem cell biology *in vivo* (Hansen and Schedl 2013; Kershner *et al.* 2013; Hubbard and Schedl 2019). In this system, germline stem cells are located at the distal end of the gonad and a single somatic cell, the distal tip cell (DTC), functions as the stem cell niche. The DTC expresses the Delta/Serrate/LAG-2 (DSL)-family ligands LAG-2 and APX-1 which act upon the Notch-family receptor GLP-1 in the germ line to promote the stem cell fate (Henderson *et al.* 1994; Nadarajan *et al.* 2009). Ablation of the DTC causes all germ cells to differentiate (Kimble and White 1981), while elevating GLP-1/Notch activity leads to a “full” tumorous phenotype in which all germ cells remain as stem cells and fail to differentiate (Berry *et al.* 1997).

A related tumorous phenotype is the proximal proliferation (Pro) phenotype. One mechanism by which this phenotype can arise is the “latent niche” mechanism (Killian and Hubbard 2005; McGovern *et al.* 2009). In this case, the activity of DSL ligands APX-1 and ARG-1 in the proximal somatic gonad inappropriately interact with GLP-1 on the surface of persistent undifferentiated germ cells and thereby drive proximal tumor formation. These ligands are expressed in cells born in late stages of somatic gonad development (Nadarajan *et al.* 2009) and act to promote ovulation (McGovern *et al.* 2018). Since initial meiotic entry (also termed “initial meiosis”; Pepper et al., 2003b)), the time when the first germ cells overtly differentiate by entering prophase of meiosis I, occurs in the third larval stage (L3), these later-appearing DSL ligands normally have no access to GLP-1-expressing germ cells. However, conditions that cause a severe delay in initial meiotic entry without interfering with the timing of proximal somatic gonad development, expose undifferentiated GLP-1-expressing germ cells to these ligands. This scenario ultimately results in a proximal germline tumor consisting of germline stem cells that never underwent differentiation (see also additional comparison with other proximal *C. elegans* germline tumors in Hubbard and Schedl 2019).

Several different cellular mechanisms can, in turn, delay initial meiotic entry. Since initial meiotic entry requires germ cells to escape the influence of DSL ligands expressed by the DTC, any conditions that interfere with this process can contribute to proximal tumor formation. Two separate processes determine the speed with which the DTC is separated from proximal-most germ cells in larval stages, namely migration of the DTC and growth of the proliferating germline stem cell pool that pushes the DTC centripetally (McGovern *et al.* 2009). Therefore, if the rate of germline proliferation is slowed such that the DTC is not pushed sufficiently centripetally by the L3/L4 molt, *proximal*-most germ cells can remain undifferentiated due to their continuous proximity to the DTC and thereby become susceptible to latent tumor-inducing signals from the proximal somatic gonad cells born in the L4 stage. Thus, somewhat counterintuitively, mutations or RNAi that interfere with robust *larval* germline proliferation can contribute to proximal tumor formation, resulting in a reduced distal adult progenitor pool and a proximal germline tumor in the same gonad arm (Killian and Hubbard 2004; Voutev *et al.* 2006).

To identify factors that predispose the germ line to proximal tumor formation, we took advantage of a temperature-sensitive weak gain-of-function (*ts-gf*) allele *glp-1**(**ar202**)*. In this mutant at the permissive temperature, proximal tumors are very rarely observed. In contrast, nearly all young adult worms shifted to the restrictive temperature as newly-hatched L1 larvae display proximal germline tumors (Pepper *et al.* 2003a). Under this condition, initial meiotic entry (Pepper *et al.* 2003b) is severely delayed, occurring in the L4 stage rather than the L3. Moreover, even though initial meiotic entry is only slightly delayed in *glp-1**(**ar202**)* mutants at the permissive temperature and worms exhibiting tumors are very rarely observed (Pepper *et al.* 2003a), proximal tumors appear frequently at the permissive temperature if initial meiotic entry is further delayed by another manipulation such as ablation of the distal-most pair of gonadal sheath cells or a reduction of insulin signaling that slows proliferation of the larval progenitor pool (Killian and Hubbard 2005; Michaelson *et al.* 2010) or by loss of *him-17* (Bessler *et al.* 2007). Therefore, this mutant is particularly sensitive to this mechanism of enhancement.

To systematically identify genetic interventions that enhance proximal tumor formation, and that may act by uncoupling the rate of germline and somatic development, we performed a genome-wide RNAi screen using a synthetic tumor formation strategy. That is, we selected genes that, when depleted by RNAi, enhanced the penetrance of proximal tumor formation in *glp-1**(**ar202**)* at the permissive temperature. Based on our previous studies (Killian and Hubbard 2005; McGovern *et al.* 2009), we sought enhancement of the penetrance of the phenotype, that is, the percentage of worms in which proximal tumors are observed under conditions in which they are otherwise rarely seen. We did not screen for changes in the size of proximal tumors, a measure of expressivity. Furthermore, our L1 RNAi feeding strategy did not exclude genes required for embryonic development. Our primary screen identified 196 unique genes, the depletion of which by RNAi caused a low-resolution “patchy” phenotype and evidence of ectopic germline proliferation, consistent with the possibility of a proximal tumor. These were further narrowed down to 43 genes that upon RNAi depletion could reproducibly enhance the penetrance of proximal tumor formation in *glp-1**(**ar202**)*, but not in *glp-1**(+)* at the permissive temperature of 15°. Few of these genes have been identified in previous screens for *glp-1**/* Notch modifiers in this system. Our list of 43 genes represent a variety of functional classes and the most enriched is “translation”, which accounts for ∼40% genes in the dataset. We further grouped these genes based on the effects of their RNAi depletion on the distal germline progenitor pool in combination with a genetic test for germline *vs.* soma autonomy.

## Materials and Methods

### Strains

All worm strains were maintained as described by Brenner (1974) and grown at 15° on NGM plates unless otherwise stated. The wild-type strain was N2 (Bristol) (Brenner 1974). Alleles used in this study were *glp-1**(**ar202**)* (Pepper *et al.* 2003a), *glp-1**(**e2141**)* (Priess *et al.* 1987; Dalfó *et al.* 2010) and *rrf-1**(**pk1417**)* (Sijen *et al.* 2001). Worm strain information can be found in the Reagent Table. The *E. coli* strains OP50 and HT115(DE3) were used for maintaining worm stocks and for RNAi, respectively.

### RNAi screening in liquid

**Figure 1:**
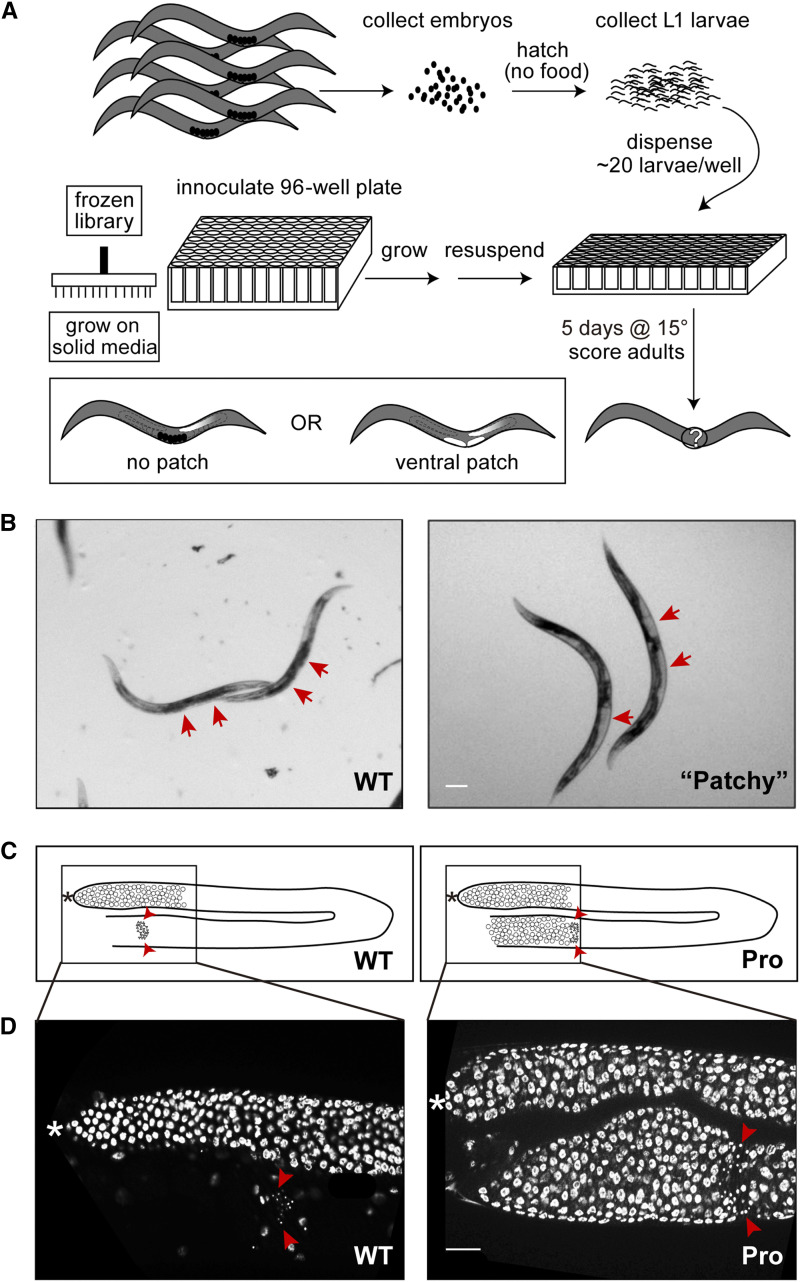
RNAi screen for enhancers of *glp-1**(Pro)*. (A) Experimental flow of the screen. Synchronized L1 larvae and RNAi-inducing bacteria were prepared in parallel and mixed in 96-well plates. The plates were incubated at 15° for five days and worms were scored at the young adult stage. Proximal tumors appeared as clear or white patches flanking the vulva. (B) Representative bright field images of young adult *glp-1**(**ar202**)* worms displaying wild-type (left) and “patchy” (right) phenotypes. Arrows point to the ventral region adjacent to the vulva. This region is dark in animals without proximal tumors due to the presence of embryos, but appears clear or white in gonad arms that contain a large proximal tumor. Note that other gonadal defects such as improper DTC migration can produce a similar ventral “patchy” phenotype when observed at low magnification (Kamath *et al.* 2003). (C) Cartoons of a wild-type (left) and a Pro (right) germ line to show where the images in (D) were taken (inner box). Note only undifferentiated germ cells and mature sperm are shown in the cartoons. (D) Representative DAPI images of young adult *glp-1**(**ar202**)* worms displaying wild-type (left) and Pro (right) phenotypes. Arrowheads point to the sperm. The proximal tumor is the mass of undifferentiated cells located proximal to sperm. Asterisk indicates the distal end of the germ line. Scale bar, 100 μm for both images in (B); 20 μm for both images in (D).

**Figure 2:**
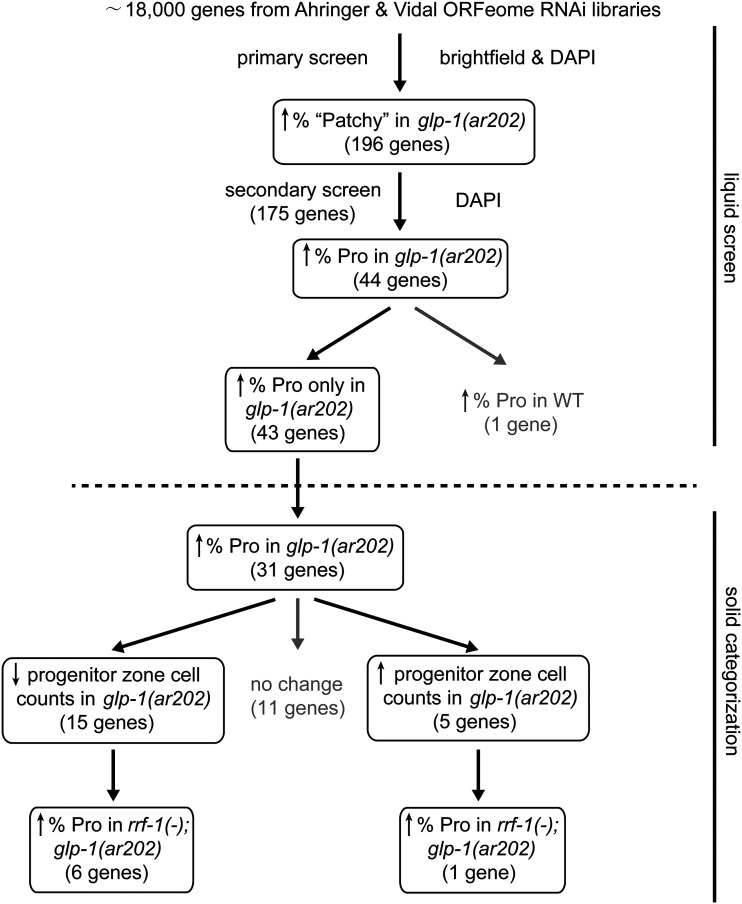
Flowchart of our RNAi screening in liquid and functional categorization after RNAi feeding on solid media. Through multiple rounds of liquid RNAi screening, we identified 43 genes that, when depleted by RNAi enhance proximal tumor formation in *glp-1**(**ar202**)*, but not in *glp-1**(+)* worms. We then grouped these genes based on results of their RNAi on solid plates by two criteria: (1) effects on the progenitor zone and (2) requirement for the soma-specific RNAi factor *rrf-1*.

The liquid RNAi-feeding was conducted essentially as described (Cipriani and Piano 2011). See Results and Figure 1 for additional primary screening details and representative images, and see Figure 2 for a flow chart of the primary and secondary screens, plus subsequent characterization of the genes identified.

Primary screening was performed using two libraries: the Ahringer library (Kamath *et al.* 2003) consisting of 16,257 bacterial clones from Geneservice Ltd and the Vidal ORFeome library (Rual *et al.* 2004) consisting of 11,559 bacterial clones from Open Biosystems. The two libraries target 9635 genes in common, and together, they target 18,181 genes (∼94% of the genome). Bacteria bearing the empty vector L4440 and an RNAi plasmid bearing DNA sequence from W07E6.2 (which was identified previously as a strong enhancer in independent unpublished experiments in the lab) were used as negative and positive controls, respectively. Parallel preparation and mixing of RNAi-inducing bacteria and hypochlorite-synchronized L1 larvae were performed as described in Roy *et al.*, (2018) (Roy *et al.* 2018). Plates containing RNAi-inducing bacteria and synchronized L1 larvae were incubated at 15° for five days in a humid chamber with agitation prior to scoring. A Leica dissecting microscope outfitted with a camera, an automated stage, and Surveyor software was used to collect one image per well. Images were scored for the percent of animals displaying discrete white or clear patches adjacent to the vulva out of total scorable animals in the same well (penetrance of “patchy”) and on a (+) to (++++) scale (+ corresponded to approximately ≤25%, ++ ∼25–50%, +++ ∼50–75%, and ++++ ≥75%). Bacterial clones in the ++, +++, and ++++ categories (a total of 457) were rescreened and 196 unique genes were identified from the primary screen. Initial analysis of these 196 genes included additional mutant backgrounds in parallel (see Results). Duplicate plates were scored for each round with approximately 20 animals per well, and the penetrance of the relevant phenotype was corrected by subtracting the corresponding penetrance of the phenotype scored in wells bearing the L4440 control bacteria for each plate (see Table S1).

A secondary screen was performed in liquid as described above. Prior to initiating the secondary screen, positive clones from the primary screen were re-isolated from the libraries and re-sequenced. This analysis revealed several discrepancies and duplications such that the secondary screen was performed on a set of clones that, after re-sequencing, were found to represent 175 unique genes. Discrepancies fell into the following categories (with the number of genes affected in parenthesis): sequence corresponded to “dead” gene in WormBase (1), different gene identity upon re-sequencing (13), and duplications in sequencing returns that eliminated a gene or genes from initial set (11). In addition, no sequence match could be obtained for 6 of the clones.

DAPI staining was performed as described previously (Michaelson *et al.* 2010). DAPI-stained worms were put on fresh 5% agar pads for visualization by fluorescence microscopy at 100x and 400x on a Zeiss Z1 AxioImager. The percent of animals bearing proximal tumors (penetrance of Pro) was scored for each RNAi treatment and Fisher’s Exact test was used to analyze the penetrance of Pro relative to the L4440 control in the secondary screen.

### Solid media RNAi and analysis of distal progenitor zone

For RNAi experiments conducted on solid plates, RNAi was carried out as described (Timmons and Fire 1998). Synchronization by L1 hatch-off, ethanol fixation, and DAPI staining were performed as described (Michaelson *et al.* 2010). In addition to proximal tumors, animals were scored for number of nuclei in the distal progenitor zone after five days at 15°. Designation of the distal progenitor zone and determination of number of nuclei in the progenitor zone were conducted as described (Korta *et al.* 2012); distal zone counts were obtained only from gonads with an unambiguous border between the progenitor zone and transition zone. Statistical analysis was done using Student’s *t*-test.

### Functional Analysis

Manual ‘Functional Class’ curation including orthologs and disease association for specific genes was performed based on WormBase gene descriptions and homology information (version WS271) and by using the Alliance of Genome Resources website (http://www.alliancegenome.org), data retrieved in July 2020. Wherever possible, functions of *C. elegans* genes were preferably used over those of their orthologs in other species. Candidate genes were grouped into the following categories: (1) Cytoskeletal: components of cytoskeleton and proteins that bind to cytoskeletal components; (2) Metabolism: enzymes involved in the synthesis, modification, and degradation of macromolecules; (3) mRNA processing: proteins involved in mRNA splicing and maturation; (4) Other: proteins with domain annotations but less clear cellular functions; (5) Proteostasis: proteins involved in protein folding and degradation; (6) Signaling: proteins involved in known cellular signaling pathways, kinases, and phosphatases; (7) Transcription: proteins that interact with the transcription machinery and that regulate gene expression at the transcriptional level; (8) Translation: components of the translation machinery as well as proteins involved in ribosome biogenesis; (9) Transport: components of ion channels and proteins involved in nuclear transport; (10) Unknown: proteins with no Pfam domain hits and no obvious orthologs outside Caenorhabditis.

Statistical overrepresentation analysis of Gene Ontology (GO) terms was performed using PANTHER Classification System v15.0 (http://pantherdb.org; (Mi *et al.* 2017; Mi *et al.* 2013)). WormBase IDs (WBGene000xxxxx) were entered for input and the Fisher’s Exact test with the default false discovery rate (FDR) calculation settings was used to determine the highly significant and enriched GO terms.

Protein functional association network analysis was performed using STRING v11.0 (https://string-db.org; (Szklarczyk *et al.* 2019)). WormBase IDs were entered for input and the default settings were used to generate an association network for proteins encoded by the genes in our dataset. Functional enrichment analysis such as the KEGG pathway analysis was also done using the default settings.

### Data availability

Strains are available upon request. Figure S1 contains a representative image of *glp-1**(+)* worms raised in the presence of *lam-1* RNAi-inducing bacteria. Table S1 contains three sheets as follows. The first is a “README”. The second sheet contains results for the last round of RNAi experiments for the 196 unique genes that were identified in the primary screen. The third sheet contains updated gene identity lists for the 196 unique genes identified in the primary screen and the 175 unique genes that were ultimately tested in the secondary screen. Table S2 contains mammalian orthologs and disease associations of the 43 genes identified in the secondary screen. Table S3 contains data for enhancement of Pro penetrance for the 31 genes whose RNAi caused elevated penetrance of Pro phenotype in *glp-1**(**ar202**)* animals on solid media. Table S4 contains data for number of progenitor zone nuclei for the 31 genes whose RNAi caused elevated penetrance of Pro phenotype in *glp-1**(**ar202**)* animals on solid media. Table S5 contains data for enhancement of Pro penetrance in *rrf-1**(**pk1417**); **glp-1**(**ar202**)* animals for the 15 genes whose normal function is required for robust distal progenitor pool expansion. Table S6 contains data for enhancement of Pro penetrance in *rrf-1**(**pk1417**); **glp-1**(**ar202**)* animals for the five genes whose RNAi caused elevated numbers of distal germline progenitor cells. Supplemental material available at figshare: https://doi.org/10.25387/g3.12420698.

## Results and Discussion

### Primary RNAi screen identified 196 genes that display a ventral “patchy” phenotype consistent with a proximal germline tumor

At low magnification, proximal tumors can appear as clear or white patches on ventral side of the worm, flanking the vulva (see Figure 1A and B). For the first round of the primary screen, we screened two commercially available RNAi libraries (Ahringer library and Vidal ORFeome library) in *glp-1**(**ar202**)* at the permissive temperature of 15° looking for ventral patches in young adult animals that were similar in appearance to patches observed in this mutant grown at the restrictive temperature of 25°.

To enable screening of genes essential for embryonic development, worms were subjected to RNAi-by-feeding starting from the first larval stage (L1) and these same animals were scored as young adults. Genes we identified remained subject to all of the usual caveats for RNAi screens such as limitations of library representation and differential RNAi responses. Nevertheless, L1 feeding effectively broadened the screen.

To improve the throughput of the screen, we performed the screen in liquid culture in duplicate 96-well plates with ∼20 animals per well (Cipriani and Piano 2011) and acquired images of each well with an automated image capture system (see Figure 1A). The time-window for optimal scoring was relatively short. This is due to the fact that proximal tumors in the oviduct are less visible at earlier time points but may be pushed into the uterus by sheath contractions and pressure from distal germ cells, thereby appearing non-tumorous at later time points. Therefore, the image capture system enabled us to improve screening throughput since many plates could be imaged at one time and scored at a later time. Each well was scored on a scale of one (+) to four (++++) representing quartiles of percentage of animals bearing the ventral “patchy” phenotype (where + corresponded to ≤25%, ++ ∼25–50%, +++ ∼50–75%, and ++++ ≥75%).

We performed a second round of the primary screen among candidates that elevated the penetrance of “patchy” >25% in the first round (groups ++, +++, and ++++; 457 clones). These were re-gridded onto new 96-well plates and retested, scoring the percentage of individual worms in each well that displayed a ventral “patchy” phenotype. In a final round of scoring, animals from each well were examined after DAPI staining and an initial assessment of the proximal proliferation (Pro) phenotype was made (see Table S1). We consider the value of this first round of DAPI analysis performed on over 450 samples as a more qualitative than quantitative assessment; the Pro phenotype was examined in more detail on a more restricted set (see below). The criteria for clones retained from this analysis of 457 clones were that they again elevated the proportion of worms displaying a “patchy” appearance and/or proximal germline tumor by DAPI (*vs.* other phenotypes that cause the patchy appearance at low magnification such as germline migration defects or generally dysmorphic gonads, *etc*.) and that a tumor phenotype was ≥10% more penetrant relative to the control. This second round of the primary screen yielded 212 RNAi clones representing 196 unique genes (see Table S1).

### Initial characterization of 196 genes from the primary screen

To obtain a first assessment of which effects may be due to RNAi action in the soma *vs.* the germ line, we analyzed the penetrance of the “patchy” phenotype for these 196 genes in the double mutant *rrf-1**(**pk1417**); **glp-1**(**ar202**)* (see Table S1). *rrf-1* encodes an RNA-directed RNA polymerase with largely soma-restricted activity. As a result, in *rrf-1* mutants RNAi remains efficient in the germ line, but is less efficient in the soma (Kumsta and Hansen 2012; Sijen *et al.* 2001). Therefore, RNAi clones that show little difference in the penetrance of the “patchy” phenotype in *glp-1**(**ar202**)* *vs.* *rrf-1**(**pk1417**); **glp-1**(**ar202**)* were candidates for germline-autonomous RNAi effects, while those with a large difference were candidates for soma-autonomous effects. Most genes showed small or intermediate differences that were difficult to interpret, especially when the “patchy” phenotype occurred at a relatively low penetrance. Nevertheless, 21 genes showed marked differences in penetrance (>75%), including *inx-8* and *inx-9*, the somatic components of gap junction channels that are required for germline proliferation (Starich *et al.* 2014), and *mpk-1* (Lee *et al.* 2007), which acts germline autonomously, is among those genes with less than 25% difference (Table S1).

One mechanism by which initial meiotic entry can be delayed is the failure to generate a robust larval germline progenitor pool (see introduction; (Killian and Hubbard 2004, 2005; McGovern *et al.* 2009; Voutev *et al.* 2006)). We reasoned that RNAi that interferes with larval germline proliferation might enhance both the penetrance of the Pro phenotype in *glp-1**(**ar202**)* and the sterility phenotype of a mutant with a reduced progenitor pool. Since the progenitor pool size correlates with fertility (Agarwal *et al.* 2018; Roy *et al.* 2018), a sub-fertile mutant subjected to RNAi that reduces progenitor production may be sterile. As an initial evaluation, we examined the effects of RNAi of the 196 genes on the temperature-sensitive loss-of-function *glp-1**(**e2141**)* mutant at the semi-permissive temperature of 20°. The progenitor pool of *glp-1**(**e2141**)* at this temperature only reaches about half the normal adult number, and therefore is sensitized to cause sterility if a given RNAi further interferes with its expansion, such that the pool stays below a threshold for timely fertility. For this analysis, we scored the percentage of worms without apparent embryos in the uterus at a time point when the controls were fertile. Although we scored this phenotype as “sterile” (*vs.* fertile/gravid), we note that slower growth of the worms due to the particular RNAi may delay progeny production. In addition, this scoring did not distinguish between those genes that, when depleted by RNAi, conferred a true “Glp-1” phenotype (see Hubbard and Schedl 2019). We found that 53 of the genes whose RNAi enhanced the “patchy/Pro” phenotype of *glp-1**(**ar202**)* in the primary screen also enhanced the percentage worms without embryos (“Sterile”) to ≥ 20% above the control in *glp-1**(**e2141**)*. This list includes several genes known to reduce the distal pool, including several involved in ribosome biogenesis such as *pro-2* (Voutev *et al.* 2006), others noted above including *inx-8**, **inx-9*, and *mpk-1* (Lee *et al.* 2007; Starich *et al.* 2014), as well as *daf-1* which was characterized in greater detail (Dalfó *et al.* 2012; Pekar *et al.* 2017).

We further examined the effect of RNAi on the distal pool in more detail on a smaller set of genes identified after the secondary screen.

### The secondary screen identified 43 genes that enhance the proximal tumor phenotype

In a secondary screen we quantitatively assayed enhancement of the Pro phenotype, as opposed to RNAi that would confer a Pro phenotype in the wild type, by comparing in parallel the effects of each RNAi on *glp-1**(**ar202**)* and on the N2 wild type (see Figure 1C and D). Prior to re-screening, we re-sequenced the 196 clones. Several discrepancies were discovered upon re-sequencing (see Methods), such that this secondary screening tested 175 unique genes (see Table S1). We found that individual knockdown of 44 out of the 175 genes caused significant elevation of the penetrance of tumor formation in *glp-1**(**ar202**)* at 15° as scored after DAPI staining (see Figure 2).

Among the 44 candidates, one gene, *lam-1*/laminin β subunit, caused penetrant tumor formation when depleted in the *glp-1**(+)* background. *lam-1* RNAi also caused rupture of gonad and escape of germ cells, a phenotype reminiscent of that caused by RNAi of *epi-1*/laminin α subunit (Gordon *et al.* 2019) (see Figure S1).

RNAi depletion of each of the remaining 43 genes caused a markedly elevated penetrance of Pro only in *glp-1**(**ar202**)*, but not in *glp-1**(+)*, and thus were carried forward for further analyses (see Figure 2, Table 1, and Table S2).

**Table 1:** List of 43 genes for which RNAi depletion caused elevated penetrance of the Pro phenotype in *glp-1(ar202)*, but not in *glp-1(+)* in the liquid RNAi screen. Gene symbol, WormBase ID, linkage group, functional class, and results of the cognate RNAi on solid plates are shown for each gene. y, yes; n, no; nd, not determined

Gene Symbol	WormBase ID	Linkage Group	Functional Class (manual)	Functional Class Notes	Elevated Pro (solid)	Altered Distal	Enh(Pro) *rrf-1*-dependent
*act-1*	WBGene00000063	V	cytoskeletal		n	nd	nd
*arf-1.2*	WBGene00000182	III	metabolism	GTP binding activity	y	↑	y
*C23G10.5*	WBGene00016012	III	unknown		n	nd	nd
*cct-7*	WBGene00020391	V	proteostasis		y	↓	y
*cdk-12*	WBGene00007135	III	transcription	phosphorylation of RNA polymerase II	y	n	nd
*cri-2*	WBGene00019478	V	proteostasis		n	nd	nd
*daf-1*	WBGene00000897	IV	signaling		n	nd	nd
*eif-3.F*	WBGene00001229	II	translation	translation initiation	y	↓	n
*F25B5.3*	WBGene00017775	III	metabolism		n	nd	nd
*F53F4.11*	WBGene00009993	V	translation	structural component of ribosome	y	↓	n
*F53H1.1*	WBGene00018776	IV	translation	rRNA modification (helicase)	y	n	nd
*fib-1*	WBGene00001423	V	translation	rRNA modification (methyltransferase)	n	nd	nd
*H06I04.3*	WBGene00019168	III	translation	rRNA modification (methyltransferase)	y	n	nd
*hda-1*	WBGene00001834	V	transcription		y	n	nd
*hoe-1*	WBGene00001983	IV	metabolism	endonuclease activity; metal ion binding activity	y	n	nd
*iff-2*	WBGene00002065	II	translation	translation initiation	y	↓	y
*knl-1*	WBGene00002231	III	cytoskeletal	microtubule binding activity	y	↑	y
*mfap-1*	WBGene00009671	I	mRNA processing	splicing	y	n	nd
*mrpl-28*	WBGene00020796	II	translation	structural component of ribosome (mitochondrial)	y	↓	y
*mrpl-4*	WBGene00020717	V	translation	structural component of ribosome (mitochondrial)	y	↓	y
*nath-10*	WBGene00018866	I	translation	rRNA modification (acetyltransferase)	y	n	nd
*nol-2*	WBGene00021073	II	translation	rRNA modification (methyltransferase)	y	n	nd
*pie-1*	WBGene00004027	III	transcription	repressor of RNA polymerase II	n	nd	nd
*pkc-3*	WBGene00004034	II	signaling/polarity		n	nd	nd
*plk-1*	WBGene00004042	III	signaling		y	n	nd
*prp-31*	WBGene00022458	I	mRNA processing		y	↓	y
*rae-1*	WBGene00003803	I	transport	nuclear export	y	↑	n
*rpl-2*	WBGene00004413	V	translation	structural component of ribosome	y	↓	y
*rpl-25.1*	WBGene00004438	X	translation	structural component of ribosome	y	↓	y
*rpoa-2*	WBGene00008781	I	translation	rRNA synthesis	y	n	nd
*rrbs-1*	WBGene00007617	V	translation	regulator of ribosome biogenesis (5S rRNA binding activity)	y	↓	n
*snr-3*	WBGene00004916	II	mRNA processing	spliceosomal snRNP assembly	y	↓	y
*spe-5*	WBGene00004959	I	transport		n	nd	nd
*sptl-3*	WBGene00011932	V	metabolism	sphingolipid metabolism	n	nd	nd
*T13H5.4*	WBGene00011758	II	mRNA processing	splicing	y	↓	y
*T26G10.1*	WBGene00012059	III	translation	rRNA modification (helicase)	y	↑	y
*tbb-1*	WBGene00006536	III	cytoskeletal		y	↑	y
*tln-1*	WBGene00006771	I	cytoskeletal	binds actin filaments	n	nd	nd
*W07E6.2*	WBGene00021074	II	translation	ribosome assembly (large subunit)	y	n	nd
*W09C5.1*	WBGene00012351	I	translation	regulator of ribosome biogenesis	y	↓	n
*xpo-1*	WBGene00002078	V	transport	nuclear export	n	nd	nd
*Y45F10D.7*	WBGene00012887	IV	other	RNA binding activity	y	↓	n
*ZK430.7*	WBGene00022742	II	signaling	estrogen receptor binding activity	y	↓	N

### RNAi enhancers of Pro encode a variety of proteins

We wished to determine which of the 43 proteins encoded by the candidate genes correspond to related human proteins. We reasoned that, despite the differences in cellular configurations that led to enhancement of proximal germline tumors in *C. elegans*, human orthologs of genes we found may contribute to Notch-related pathologies (Siebel and Lendahl 2017). Using gene descriptions and homology information on WormBase (version WS271) and the Alliance of Genome Resources database, we found 40 out of the 43 *glp-1**(Pro)* enhancers have easily-identified human orthologs, and 15 have clear disease associations (Table S2).

**Figure 3:**
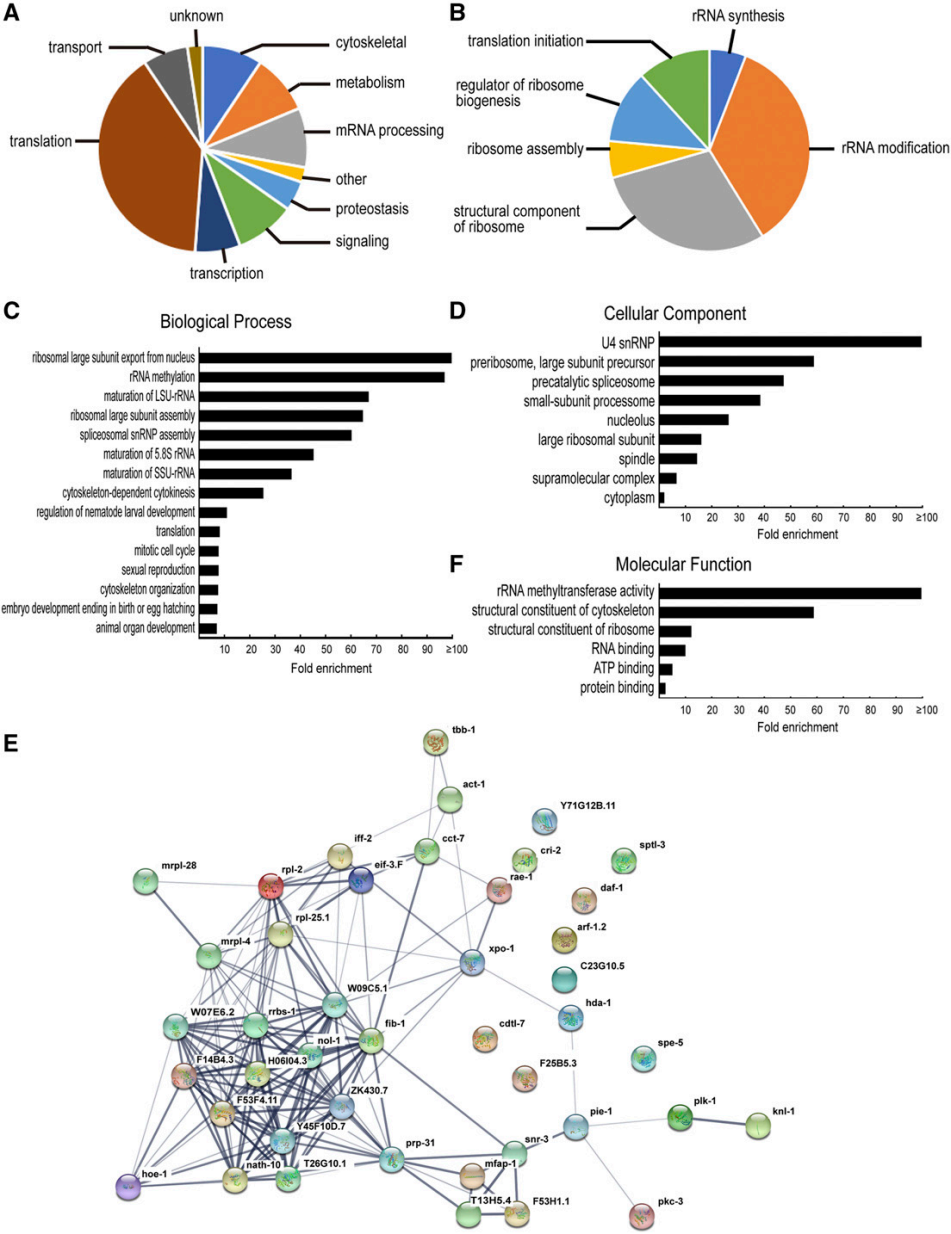
Functional classification of the 43 genes identified in the liquid RNAi screen. (A) Manual classification of candidate genes using gene descriptions and homology information on WormBase and the Alliance of Genome Resources database. We defined “cytoskeletal” as components of cytoskeleton and proteins that bind to cytoskeletal components; “metabolism” as enzymes involved in the synthesis, modification, and degradation of macromolecules; “mRNA processing” as proteins implicated in mRNA splicing and maturation; “Other” as proteins with domain annotations but less clear cellular functions; “proteostasis” as proteins involved in protein folding and degradation; “signaling” as proteins implicated in known cellular signaling pathways, kinases, and phosphatases; “transcription” as proteins that interact with the transcription machinery and that regulate gene expression at the transcriptional level; “translation” as components of the translation machinery as well as proteins involved in ribosome biogenesis; “transport” as components of ion channels and proteins implicated in nuclear transport; “Unknown” as proteins with no Pfam domain hits and no obvious orthologs outside Caenorhabditis. (B) “translation” in (A) is divided into several subclasses. (C-E) Statistical overrepresentation analysis of Gene Ontology (GO) terms using the PANTHER Classification System. The most specific subclass of each of the significantly enriched Biological Process (BP), Cellular Component (CC), and Molecular Function (MF) classes are shown in (C), (D), and (E), respectively. (F) A functional association network generated by the STRING website. Network nodes represent proteins and edges represent protein-protein associations. Line thickness indicates the strength of data support for a particular interaction.

We also manually curated the 43 candidate genes based on their molecular nature and the cellular processes they are involved in, and we classified them into 10 categories. Among them, “translation” was the most abundant group, consisting nearly 40% of the genes on the list, followed by “cytoskeletal” (four), “metabolism” (four), “mRNA processing” (four), “signaling” (four), “transcription” (three) and “transport” (three), whereas “proteostasis” (two), “other” (one) and “unknown” (one) were the least represented groups (Figure 3A).

To determine which functional classes are overrepresented in our dataset relative to the *C. elegans* genome, we performed a statistical overrepresentation test of Gene Ontology (GO) terms associated with those 43 genes using PANTHER Classification System v15.0 (Mi *et al.* 2017; Mi *et al.* 2013). PANTHER recognized all 43 genes and identified 15 significantly enriched Biological Process (BP), nine Cellular Component (CC), and six Molecular Function (MF) classes. “Ribosomal large subunit export from nucleus (GO:0000055)” and “rRNA methylation (GO:0031167)” had the highest fold enrichment (>100 and 97.21, respectively) compared to the *C. elegans* reference genome among the BP classes (Figure 3C). “U4 snRNP (GO:0005687)” and “rRNA methyltransferase activity (GO:0008649)” were the most overrepresented CC and MF classes, respectively (both fold enrichment >100, Figure 3D and E). These data are consistent with results from our manual classification, since genes in the above classes were grouped into “translation” and “mRNA processing”, the most abundant categories in our manual analysis.

We also conducted “protein functional association network” analysis using STRING v11.0 (https://string-db.org; (Szklarczyk *et al.* 2019)). This analysis generated a functional association network for proteins encoded by the 43 genes in our dataset. Protein-protein interactions with high confidence were observed among proteins that were classified into the “translation” category in our manual analysis (see Figure 3F). Furthermore, STRING analysis also identified “ribosome biogenesis in eukaryotes” as the most overrepresented KEGG pathway of our gene list.

Previous analyses implicated several genes involved in ribosome biogenesis in distal sheath development and in the expansion of the larval germline progenitor pool, leading to the Pro phenotype when their function was compromised (Killian and Hubbard 2004; Voutev *et al.* 2006). Although our screen only identified one of the previously implicated factors (*W07E6.2*), the identification of a cluster of genes involved in many different steps of ribosome biogenesis/translation is intriguing. Our screen identified seven enzymes involved in rRNA synthesis and modification (one RNA polymerase I subunit, two RNA helicases, one rRNA acetyltransferase, and three rRNA methyltransferases), five structural components of ribosome (including two of mitochondrial ribosome), one protein implicated in ribosome assembly, two regulators of ribosome biogenesis, and two translation initiation factors (see Figure 3B). These genes consist almost 40% of the candidates identified in our screen, suggesting that defects in ribosome biogenesis/translation are a major cause for enhancement of the Pro phenotype in *glp-1**(**ar202**)* at the permissive temperature.

Many genes throughout the splicing cascade have been implicated in germline proliferation, including several pre-mRNA processing factors (*mog-1*, *mog-4*, *mog-5*, *ddx-23*, *prp-17*, *teg-1*, and *teg-4*). A decrease in these genes’ function often results in reduced distal germline proliferation and enhancement of proximal tumor formation (Belfiore *et al.* 2002; Kerins *et al.* 2010; Konishi *et al.* 2008; Mantina *et al.* 2009; Puoti and Kimble 1999, 2000; Wang *et al.* 2012; Wang *et al.* 2014; Zanetti *et al.* 2011). Indeed, our screen identified four splicing factors, two of which (*prp-31* and *T13H5.4*) overlap with those identified in a screen of pre-mRNA processing factors for synthetic tumor formation in another sensitized background (*rrf-1**; **gld-3**)* (Kerins *et al.* 2010). It is not yet known which exact pre-mRNA substrates of these factors might be important to ensure timely initial meiotic entry, or if these factors may be alternatively required to stabilize RNP complexes that are necessary for timely germline development.

Enhancer and suppressor screens of *C. elegans* Notch receptor mutants have identified core components and modifiers of Notch signaling, and the outcome of a particular screen largely depends on the specific allele used and the cellular context of the screen. Our screen did not identify any of the known core components of the Notch signaling pathway, nor any of the 37 well characterized modulators of *glp-1* and *lin-12* activity (*lin-12* is the other Notch receptor in *C. elegans*), as recently reviewed (Greenwald and Kovall 2013; Hubbard and Schedl 2019).

We also compared our set of 43 genes with the 483 genes that have predicted or experimentally confirmed interactions with *glp-1* (including genetic, regulatory, and physical interactions) as listed in WormBase (WS271). Four genes (*hda-1*, *pie-1*, *plk-1*, and *prp-31*) were shared. Thus, our study provides 39 additional candidates for functional interactions with *glp-1*/Notch, some of which likely enhance the Pro phenotype as a result of general effects on the timing of germline development. Nevertheless, this phenotypic interaction can serve as an entry point for future functional analysis of these genes.

As noted above, inadequate larval progenitor zone expansion can enhance both *glp-1**(**ar202**)* and *glp-1**(**e2141**)*. Indeed, 9 of the 43 genes we identified were among our potential enhancers of *glp-1**(**e2141**)* (Table S1). Two additional genes in our set of 43, *F53F4.11* and *mrpl-4*, overlap with those in an RNAi screen for “germline-specific” enhancers of *glp-1**(**e2141**)* (Roy *et al.* 2018).

Several additional genes emerged from previous screens and subsequent characterization as enhancers of *glp-1**(**ar202**)*. These include *teg-4**, **teg-1* (as noted above), *pas-5**, **pbs-4**, **rfp-1* and *kin-10* (Gupta *et al.* 2015; Macdonald *et al.* 2008; Mantina *et al.* 2009; Wang *et al.* 2012; Wang *et al.* 2014). None of these genes are in our set of 196 or 175 (however, see below regarding *kin-3* and *kin-10* that encode subunits of casein kinase II). One possible explanation for the difference is that these screens used different starting strains (*e.g.*, *gld-3*) and/or different RNAi conditions (*e.g.*, maternal RNAi rather than L1 feeding).

Among the 43 genes in this final set of candidates is *daf-1*/TGFβRI, which was selected for analysis, together with *daf-7*, from the primary screen candidates. These genes and others in the *daf-7*/TGFβ pathway have been implicated in germline progenitor development (Dalfó *et al.* 2012; Pekar *et al.* 2017). While *daf-7* itself was in the lists of 196 and 175, this gene did not make the more stringent list of 43, possibly due to a weaker and more variable effect of RNAi in neurons. Similarly, *kin-3*, a casein kinase II alpha ortholog, was in the larger sets but not the more stringent set of 43. Casein kinase II beta, *kin-10*, was identified independently in an RNAi screen for enhancers of *gld-3*, and was shown to enhance *glp-1**(**ar202*) (Wang *et al.* 2014). This suggests that other genes on the larger lists that did not make our most stringent tests may also prove relevant.

Our analysis also identified two genes, *C23G10.5* and *Y45F10D.7*, whose cellular function is so far completely unknown. Therefore, our screen provides a first phenotype that could be used to further functionally characterize them.

### A subset of genes identified in the liquid RNAi screens enhance the Pro phenotype by RNAi on solid media

We further tested the 43 candidate genes identified in our liquid screen in an RNAi feeding strategy on solid plates. Not surprisingly, due to differences in growth conditions that may affect RNAi efficacy (*e.g.*, worms feeding on abundant bacteria on solid plates *vs.* in liquid where feeding is more difficult and worms are constantly swimming), only a subset of those genes (31 out of 43) enhanced proximal tumor formation in *glp-1**(**ar202**)* when retested at the permissive temperature of 15° with RNAi bacteria on solid plates (see Figure 2 and Table S3).

### The distal progenitor pool in early adult glp-1(ar202) is altered by RNAi of 20 genes

**Figure 4:**
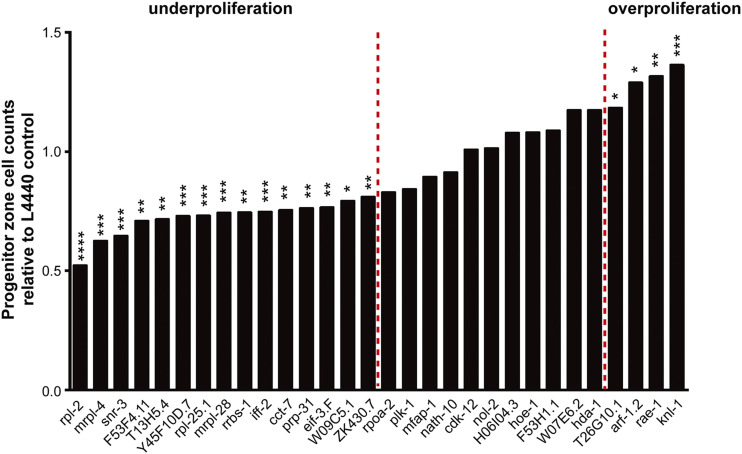
Distal progenitor zone cell counts of young adult *glp-1**(**ar202**)* worms upon RNAi depletion of 30 individual genes relative to the L4440 control. Red dotted lines mark the *P* ≤ 0.05 statistical cutoff. *glp-1**(**ar202**)* worms treated with RNAi of genes on the left side of the left dotted line had significantly fewer cells in the distal progenitor pool, whereas RNAi of genes on the right side of the right dotted line caused accumulation of excessive progenitors. *tbb-1* is not shown because “tumorous” growth in the distal germ line of *tbb-1* RNAi treated animals precluded an accurate assessment of the number of their progenitors. n = 8-10 for each RNAi condition. *, *P* < 0.05; **, *P* < 0.01; ***, *P* < 0.001; ****, *P* < 0.0001 for Student’s *t*-test.

We further analyzed these 31 genes. First, we categorized them based on the effect of RNAi on the distal germ line. We reasoned that different effects on the progenitor zone (either limiting or expanding the zone) may suggest distinct causes of the “enhancement of Pro” phenotype. For example, a reduced distal progenitor pool suggests that the target gene is required for robust larval progenitor pool expansion, whereas accumulation of excessive progenitors might result from elevated GLP-1 signaling. Among the 31 genes, RNAi of 20 altered the size of the distal progenitor pool of *glp-1**(**ar202**)* animals while 11 did not change the number of cells in the progenitor zone in a statistically significant manner. Specifically, *glp-1**(**ar202**)* animals treated with RNAi of 15 of the 31 genes had significantly fewer progenitors compared with the empty vector RNAi control, whereas animals treated with RNAi depleting five other genes had considerably greater numbers of progenitors (see Figure 4 and Table S4). Therefore, our screen identified factors with opposite effects on the distal germ line of *glp-1**(**ar202**)*.

To further explore how germline *vs.* soma autonomy may link changes in the distal progenitor pool to enhancement of the Pro phenotype, we reexamined the enhancement of Pro phenotype in these 20 genes that displayed changes in the distal germ line of *glp-1**(**ar202**)* worms in *rrf-1**(**pk1417**); **glp-1**(**ar202**)* and asked whether a subset of the RNAi enhancement phenotypes we observed were dependent on *rrf-1*. No significant enhancement of Pro phenotype in *rrf-1**(**pk1417**); **glp-1**(**ar202**)* was observed for 13 of the 20 genes upon RNAi depletion, suggesting that the functions of these genes that are relevant to the formation of proximal tumors are not solely required in the germ line. We also noted that there is no general correlation between *rrf-1* requirement and roles in the distal progenitor zone, as genes that have similar effects on germline progenitor accumulation do not share the same requirement for *rrf-1* (see Tables S5 and S6).

However, we did observe some interesting correlations between the effect on the distal progenitor pool and *rrf-1*-dependence for genes representing subclasses of the “translation” functional group. First, although genes involved in many different stages of ribosome biogenesis/translation enhanced proximal tumor formation when depleted in *glp-1**(**ar202**)* animals, their effects on the distal progenitor pool showed clear separation. RNAi of genes implicated in rRNA synthesis and modification resulted in largely normal distal progenitor zone (although a slight increase in the number of cells in the progenitor zone that barely made it to the significance cutoff was seen with *T26G10.1* RNAi), while intact function of structural components of the ribosome, regulators of ribosome biogenesis, and translation initiation factors are required for robust expansion of the progenitor zone. Second, within the latter group, regulators of ribosome biogenesis seem to act germline autonomously, as their RNAi still caused enhancement of Pro phenotype in the *rrf-1**(**pk1417**); **glp-1**(**ar202**)* animals, whereas most structural components of ribosome (5 out of 6) required *rrf-1* activity to influence proximal tumor formation, indicating a likely germline non-autonomous role for these genes. Together, these results suggest that distinct underlying mechanisms might be present for different subclasses of the “translation” functional group to affect proximal tumor formation.

We also noted a possible correlation between effects on germline progenitors and *rrf-1* requirement for genes in the “mRNA processing/splicing” functional category. While acknowledging the caveat that loss of *rrf-1* may enhance germline RNAi efficacy of these genes, RNAi depletion of three out of four genes in the “mRNA processing/splicing” group resulted in underproliferation of the distal germ line and their enhancement of proximal tumor formation required *rrf-1* activity.

Altogether, our results highlight multiple genes in a variety of functional classes that influence both the number of progenitors in the distal germ line as well as the propensity to form proximal tumors in the *glp-1**(**ar202**)* mutant background. Further, they provide initial observations for deciphering the underlying mechanisms of their activity in the control of larval germline development in *C. elegans* as well as for considering their implications for related proteins in Notch-regulated processes in other organisms.
